# Hydrogen Induced Abrupt Structural Expansion at High Temperatures of a Ni_32_Nb_28_Zr_30_Cu_10_ Membrane for H_2_ Purification

**DOI:** 10.3390/membranes6040048

**Published:** 2016-11-21

**Authors:** Oriele Palumbo, Francesco Trequattrini, Madhura Hulyalkar, Suchismita Sarker, Narendra Pal, Dhanesh Chandra, Ted Flanagan, Michael Dolan, Annalisa Paolone

**Affiliations:** 1 Consiglio Nazionale delle Ricerche, Istituto del Sistemi Complessi, U.O.S. La Sapienza, Piazzale A. Moro 5, Rome 00185, Italy; oriele.palumbo@roma1.infn.it (O.P.); francesco.trequattrini@roma1.infn.it (F.T.); 2 Department of Physics, Sapienza University of Rome, Piazzale A. Moro 5, Rome 00185, Italy; 3 Department of Chemical and Materials Engineering, University of Nevada, Reno, NV 89557, USA; hulyalkar.madhura@gmail.com (M.H.); suchismita13994@gmail.com (S.S.); narendrakpal@gmail.com (N.P.); dchandra@unr.edu (D.C.); 4 Department of Chemistry, University of Vermont, Burlington, VT 05405, USA; Ted.Flanagan@uvm.edu; 5 Commonwealth Scientific and Industrial Research Organisation, Queensland Centre for Advanced Technologies, Energy, 1 Technology Court, Pullenvale 4069, Australia; Michael.Dolan@csiro.au

**Keywords:** lattice expansion, amorphous membranes, hydrogen sorption, XRD, DTA, hydrogenation enthalpy, *61.05.cp*, *61.43.Dq*, *65.60.+a*

## Abstract

Ni-Nb-Zr amorphous membranes, prepared by melt-spinning, show great potential for replacing crystalline Pd-based materials in the field of hydrogen purification to an ultrapure grade (>99.999%). In this study, we investigate the temperature evolution of the structure of an amorphous ribbon with the composition Ni_32_Nb_28_Zr_30_Cu_10_ (expressed in atom %) by means of XRD and DTA measurements. An abrupt structural expansion is induced between 240 and 300 °C by hydrogenation. This structural modification deeply modifies the hydrogen sorption properties of the membrane, which indeed shows a strong reduction of the hydrogen capacity above 270 °C.

## 1. Introduction

For purification of hydrogen to an ultrapure grade (>99.999%), crystalline Pd and Pd-Ag (100–200 μm thickness) membranes have been employed for several decades [1,2]. Usually, one side of the membrane is exposed to the pressure of a gas containing H_2_. The membrane splits the hydrogen molecule, and the atomic hydrogen is absorbed in the metal and diffuses towards the lower H_2_ concentration side. On the low-pressure face, the hydrogen atoms recombine to form H_2_ molecules and pure hydrogen is released thanks to the high selectivity of the metallic membranes. For the Pd-based membranes, H_2_ flux values in excess of 1 mol·m^−2^·s^−1^ have been reported [3].

However, Pd is an expensive material and in certain conditions can suffer from embrittlement, due to the formation of hydrides. In order to prevent embrittlement, alloying of Pd with Ag, Au or Ru is usually performed [4]. To mitigate the economic impact of Pd, thin layers of Pd-Ag alloys (about 50 μm) were shown to have both complete selectivity to hydrogen and good durability [5]. More recently, a different approach was proposed, in which small particles of Pd (200 nm) were mixed with silica substrates [6]. Nevertheless, palladium is also a strategic material and, therefore, alternative materials or novel geometries for hydrogen separation membranes are under consideration. Among them, separation by means of polymeric membranes is of great interest [7,8,9]. Gas separation by means of polymer membranes is already used in industrial processes, such as the production of technical-grade nitrogen, hydrogen separation in refineries and the petrochemical industry and the separation of CO_2_ from CH_4_ or N_2_ [8]. In these materials, the H_2_ molecules do not need to split into hydrogen atoms to diffuse through the polymeric membranes [9]. Polymer membranes can be used for gas separation in the temperature range below 100 °C and, generally speaking, they are relatively cheap; however, usually their selectivity is relatively low and these membranes suffer from swelling and poisoning [1]. Another concern about polymer membranes is the need to recover the toxic by-products for their production [10].

Other classes of separation membranes are those based on ceramics or on porous carbon. The last one is brittle and has a selectivity for hydrogen in the range of 4–20 [1]. Also, ceramic membranes are brittle and in their dense form can be used above 600 °C [1]. An important part of the research on hydrogen purification membranes deals with the use of other metallic materials. Indeed, some metals show equivalent or greater permeability than Pd [11]. Furthermore, many of these metals, such as Ni, Co, Nb, and Zr, cost in the range of 5–40 USD·kg^−1^, making them of great interest as potential Pd alternatives. Among other options, amorphous alloys formed from a combination of Ni and one or more early transition metals show great potential for this application [12]. Due their low cost and high selectivity, amorphous membranes have been largely investigated. A review of the properties of amorphous alloy membranes for hydrogen purification is reported in Reference [13]. Amorphous alloy membranes can be produced by different methods, such as arc-melting, die-casting and melt-spinning [1]. Here, we will focus on ribbons formed directly by melt-spinning, i.e., by rapid solidification on the surface of a rolling copper wheel (ΔT/Δt ~ 10^5^ °C·s^−1^). One of the main concerns about such membranes is the possible crystallization during operation at high temperature, because it leads to the reduction in hydrogen diffusion pathways and often induces brittleness in the ribbons. Therefore, care should be used to avoid operation close to the crystallization temperature. In this framework, knowledge of the temperature evolution of the structure of the amorphous membranes is extremely important.

A promising class of amorphous alloy membranes is the Ni-Zr or Ni-Nb-Zr alloys. Hara et al. reported that Ni-Zr-based alloys are stable and do not become brittle in the temperature range between 200 and 350 °C while the hydrogen permeability of the Ni_64_Zr_36_ sample is on the order of 10^−9^ mol·m^−1^·s^−1^·Pa^−0.5^ [14,15,16]. Moreover, the hydrogen permeability of Ni-Nb-Zr amorphous samples is significantly enhanced by the addition of Zr to the alloys and it is comparable to that of the Pd-based alloys, i.e., 10^−9^–10^−8^ mol·m^−1^·s^−1^·Pa^−0.5^ [17,18,19,20,21]. The crystallization temperature of Ni-Nb-Zr alloys is generally higher than 450 °C [22,23], the maximum temperature at which permeation tests are usually conducted. These amorphous membranes can be used in the temperature range of around 400 °C, compatible with the water shift reaction of syn-gas [13].

From a structural point of view, Ni-Nb-Zr membranes are quite complex. In their amorphous state, they are composed of icosahedral clusters with a random distribution of the three chemical elements on the vertexes [18,24,25]. Density functional theory (DFT) calculations indicate that Ni-centered clusters have lower energy than the Nb- or Zr-centered clusters and, moreover, clusters with smaller Nb-Ni coordination numbers have lower energy [25]. Additional DFT calculations and the comparison with EXAFS (extended X-ray absorption fine structure) and XANES (X-ray absorption near edge structure) data suggest that the most stable hydrogen site is formed by two tetrahedra consisting of two Zr and two Nb atoms. The next stable site is formed by two tetrahedra consisting of three Zr atoms and one Nb atom [24]. Moreover, EXAFS measurements suggest that the Zr-Zr distance drastically increases upon hydrogenation and that hydrogen may easily permeate between the Zr-Zr pairs where the Zr-Zr bond length is expanded by the hydrogen atoms, giving rise to the high permeability [18]. Yamaura et al. [18] therefore suggested a link between the structural and functional properties of Ni-Nb-Zr membranes. Moreover, in the hydrogenated state some nanometric islands of ZrH_2_ can be formed [23,26,27]. Finally, starting from the amorphous phase, heating the sample above the crystallization temperature, usually more than one crystalline phase is formed [23].

Furthermore, Yamaura et al. [28] investigated the changes induced by the addition of a different element in the Ni-Nb-Zr ternary alloys on hydrogen permeability; among all the considered elements (Al, Co, Cu, P, Pd, Si, Sn, Ta and Ti), the best permeability results were obtained by the copper addition. The copper substitution does not increase the crystallization temperature, which is considered a key parameter for the potential use of these membranes; however, for (Ni_0.6_Nb_0.4_)_45_Zr_50_Cu_5_ alloy, the copper substitution increases the permeability significantly. Yamaura et al. [28] showed that hydrogen permeability decreases with the increasing Vickers hardness and concluded that the Ni-Nb-Zr-X (X = Co or Cu) amorphous alloys have high potential as hydrogen-permeable membranes. Indeed, at 400 °C the permeability increases from 0.11–1.30 × 10^−8^ mol·m^−1^·s^−1^·Pa^−0.5^ for (Ni_0.6_Nb_0.4_)_50_Zr_50_ to 2.34 × 10^−8^ mol·m^−1^·s^−1^·Pa^−0.5^ for (Ni_0.6_Nb_0.4_)_45_Zr_50_Cu_5_, which is comparable to the permeability of conventional Pd-Ag alloys.

In order to obtain materials with high permeability but low hydrogen embrittlement, knowledge of their hydrogenation properties is important as it can provide information about the hydrogen effect on the material structure. In this framework, in this paper, we will report a study of the structure of a Ni_32_Nb_28_Zr_30_Cu_10_ amorphous membrane, performed between 25 and 400 °C, by means of high temperature XRD measurements (HTXRD), either in a hydrogen or in a helium atmosphere. We will show that upon hydrogenation, the membranes display an abrupt expansion of the structure around 300 °C. In correspondence with this structural modification, the hydrogen absorption properties change and the membrane absorbs much less hydrogen in the high temperature regime than in the low temperature one.

## 2. Results and Discussion

### 2.1. Preliminarily DTA Measurements

**Figure 1:**
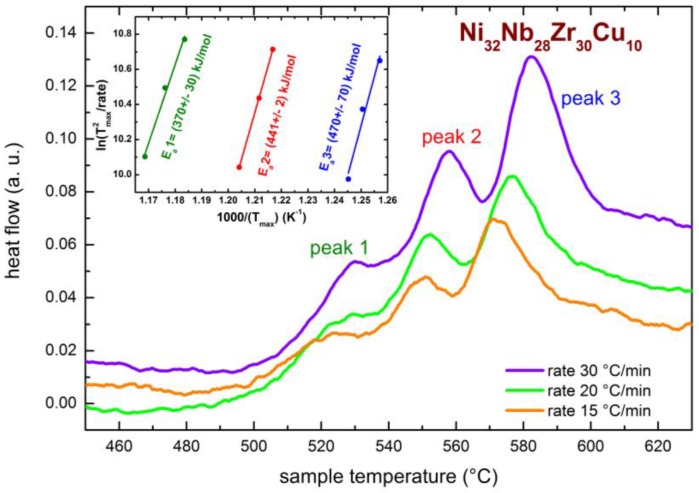
DTA signal measured at different heating rates. The inset reports the Kissinger plot for the three thermally activated peaks shown by the DTA curves.

As the occurrence of crystallization changes the absorption properties of the Ni-Nb-Zr membranes, we preliminary investigated the crystallization process by DTA measurements on the Ni_32_Nb_28_Zr_30_Cu_10_ sample. In the similar compound Ni_42_Nb_28_Zr_30_, which does not contain Cu, it has been reported that crystallization occurs at temperatures higher than 480 °C [20,23,29]; however, an investigation of the presently investigated composition is still missing. Figure 1 reports the DTA curves measured at different temperature rates, ranging between 15 and 30 °C/min.

They present three subsequent thermally activated peaks, starting above 480 °C, centered around 520, 550 and 570 °C, when measured at ΔT/Δt = 15 °C/min. The inset of Figure 1 displays the Kissinger plot for the three peaks. The calculated activation energies of the three processes are 370 ± 30, 441 ± 2 and 470 ± 70 kJ/mol. Such values are on the same order of magnitude of those already reported for other Ni-Nb-Zr membranes [20,22,23,29]. The DTA measurements indicate that the crystallization process, occurring in subsequent steps, starts above 480 °C.

### 2.2. Hydrogen Absorption Properties

The hydrogen absorption properties of the membranes were measured by obtaining pressure-composition isotherms at different temperatures in the range between 158 and 400 °C; these temperatures are well below the crystallization temperature obtained by the previously reported DTA experiments.

**Figure 2:**
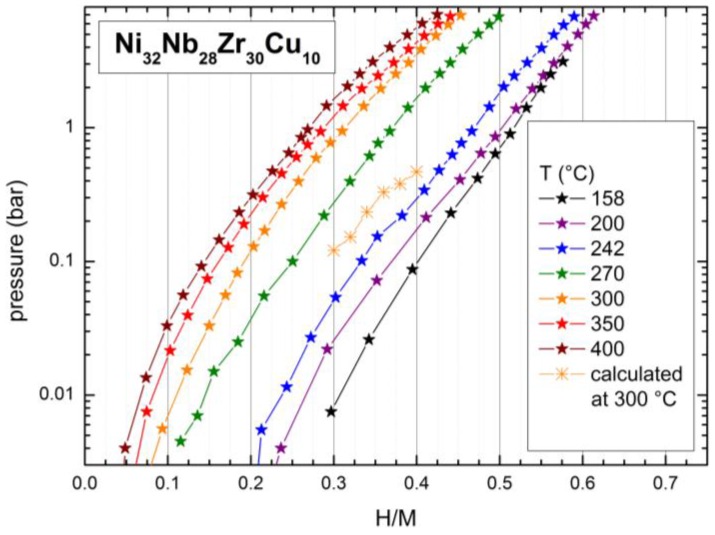
Pressure-composition isotherms measured at various temperature between 158 and 400 °C.

The pressure-composition isotherms of the Ni_32_Nb_28_Zr_30_Cu_10_ membrane are reported in Figure 2. We indicate the hydrogen concentration in the sample as H/M, i.e., the ratio between the number of hydrogen atoms and the number of metal atoms. The measurements at low temperatures were possible thanks to the fast kinetics of the sorption process in this membrane (at least 10 times faster than in Ni_42_Nb_28_Zr_30_). Figure 2 shows the absence of any pressure plateau in the whole temperature range. A similar behavior has been already observed for Ni-Nb-Zr amorphous ribbons [29]. Pressure plateaus are, on the contrary, typically observed in crystalline hydrides. Moreover, the hydrogen capacity of the Ni_32_Nb_28_Zr_30_Cu_10_ membrane is higher at lower temperatures or, equivalently, the hydrogen solubility at a fixed p decreases as T increases; for example, for p = 1 bar one observes a hydrogen content of ~0.52 H/M at 158 °C and only ~0.27 H/M at 400 °C. Some single points along the pressure-composition isotherms were duplicated in order to verify the reproducibility of the measurements.

Only a limited comparison of the hydrogen absorption properties of the presently investigated Ni_32_Nb_28_Zr_30_Cu_10_ membrane with the previous literature is possible. Indeed, in many cases Ni-Nb ribbons were hydrogenated by means of electrolytic charging [30,31], instead of the direct reaction with H_2_ gas. Conic et al. [32] measured only the absorption kinetics at various temperatures of Zr alloys containing Nb and Ta mixtures (10 wt % Nb, 12 wt % Ta and 10 wt % Nb and 12 wt % Ta). Hao et al. reported pressure-composition curves measured at 300 °C for Ni_60_Nb_40_, (Ni_0.6_Nb_0.4_)_50_Zr_50_ and (Ni_0.6_Nb_0.4_)_70_Zr_30_ [33]. The hydrogen content increases as the Zr content increases, reaching p ≈ 0.5 MPa ~1.1 mass % in (Ni_0.6_Nb_0.4_)_50_Zr_50_ and ~0.7 mass % in (Ni_0.6_Nb_0.4_)_70_Zr_30_ [16]. Also Yamaura et al. measured a single pressure-composition curve of (Ni_0.6_Nb_0.4_)_70_Zr_30_ at 300 °C [18]. Recently, we investigated the sorption properties of melt-spun (Ni_0.6_Nb_0.4−*y*_Ta_y_)_100−*x*_Zr*_x_* with *y* = 0, 0.1 and *x* = 20, 30, between 300 and 400 °C [29]. Compared to (Ni_0.6_Nb_0.4_)_70_Zr_30_, the presently investigated membranes display a higher solubility by ~0.02 H/M at 300 °C and ~0.04 H/M at 400 °C.

**Figure 3:**
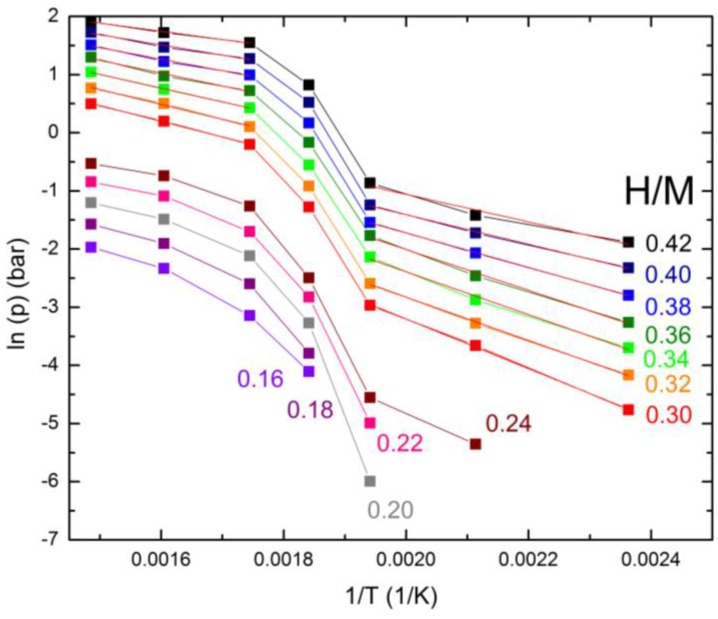
Van’t Hoff plot for the Ni_32_Nb_28_Zr_30_Cu_10_ membrane calculated at different H/M ratios and best fit lines in the low- and high-temperature range.

The sorption isotherms reported in Figure 2 are almost parallel to one another; however, one can observe a large displacement between the curve corresponding to 242 °C and that corresponding to 270 °C, and between the latter one and the isotherm measured at 300 °C, while all other curves display a constant shift toward lower H/M values as T increases (see afterwards for a quantitative analysis). Such a change in the absorption properties of the membranes is even more evident considering the van’t Hoff plot, as reported in Figure 3.

In the case of crystalline materials, for which a pressure plateau is observed in the p-c isotherms, the van’t Hoff plot reports ln(p) vs. 1/T, where p is the plateau pressure at temperature T (K) [26], and the slope of the best fit lines provides the hydrogenation enthalpy, ΔH_hyd_. However, the pressure-composition isotherms of the presently studied amorphous materials do not exhibit any plateau. In this case, one can still construct a van’t Hoff plot reporting ln(p) vs. 1/T, where p is the pressure at a fixed value of H/M at the various temperatures. Also, in this case, one can estimate the hydrogenation enthalpy, ΔH_hyd_, from the slope of the plot [34]. In order to obtain the values of p at fixed values of H/M at the various temperatures, an interpolation of the experimental data using beta splines was performed by means of a program implemented with the Labview routines. The van’t Hoff plot for the Ni_32_Nb_28_Zr_30_Cu_10_ membrane calculated for H/M from 0.30 to 0.42 in steps of 0.02 is reported in Figure 3, together with the best fit lines.

Figure 3 clearly shows two different slopes, one in the temperature range between 158 and 242 °C and another between 300 and 400 °C; however, an evident jump is present between 242 and 300 °C. Even the hydrogenation enthalpy of the material changes when passing from the low T region to the high temperature one. For example, for H/M = 0.30, ΔH_hyd_ passes from 36 ± 1 kJ/mol at low T to 23 ± 1 kJ/mol at high temperature. It can be noted that ΔH_hyd_ depends on the particular H/M considered, as reported in Table 1.

The dependence of the hydrogenation enthalpy on the hydrogen content has already recently been reported for some other amorphous membranes for hydrogen purification, namely (Ni_0.6_Nb_0.4−*y*_Ta_y_)_100−*x*_Zr*_x_* (*x* = 0.2, 0.3; *y* = 0, 0.1) membranes [29]. In the case of (Ni_0.6_Nb_0.4_)_70_Zr_30_ the enthalpy decreases from 41 kJ/mol for H/M = 0.34 to 33 kJ/mol for H/M = 0.42 [29]. This trend was attributed to the different interstitial sites available for hydrogen trapping during the progression of the absorption process: at the beginning, the deepest energy levels are occupied, while only shallower energy levels are available at the higher hydrogen content [29].

For the presently studied Ni_32_Nb_28_Zr_30_Cu_10_ membrane, the calculated ΔH_hyd_ both in the lowtemperature and in the high T regions decreases as H/M increases, similar to the other (Ni_0.6_Nb_0.4−*y*_Ta_y_)_100−*x*_Zr*_x_* membranes [29]. However, for the low temperature phase, ΔH_hyd_ is much higher than that of the high temperature phase, showing the different sorption properties of the compound in the two temperature ranges. Indeed, in Figure 2 we report a piece of the p-c curve at 300 °C calculated as the extrapolation of the low temperature curves, i.e., using ΔH_hyd_ obtained for the low T region. It is well evident that the experimental curve is shifted to a lower H/M by about 0.2–0.23.

The observed change of the absorption properties around 240 °C could be related to a structural modification of the sample. However, the membrane should retain its amorphous structure, at least within the detection limit of the previously reported DTA measurements, which do not show any peak in this temperature range. To further study this hypothesis, we performed an XRD study of the high temperature evolution of the sample structure.

### 2.3. XRD Study of the High Temperature Evolution of the Structure

**Figure 4:**
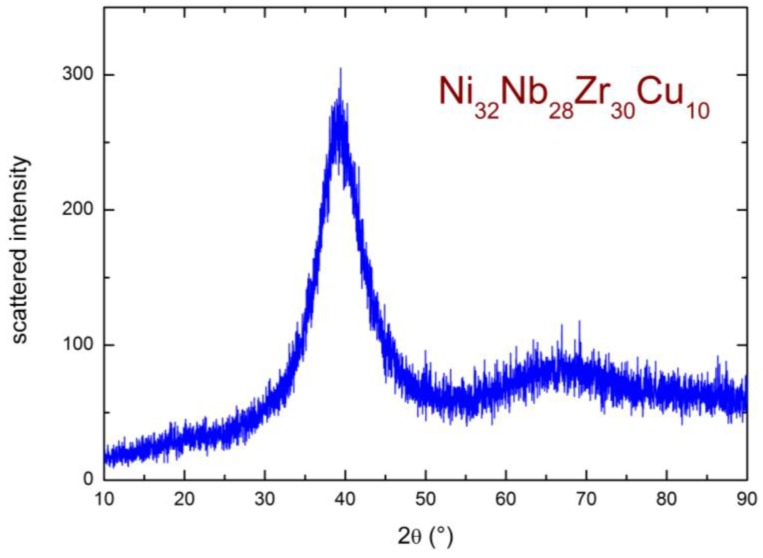
XRD spectrum of the pristine Ni_32_Nb_28_Zr_30_Cu_10_ membrane measured at room temperature in a He atmosphere.

The XRD pattern of the pristine membrane, measured at room temperature in a helium atmosphere, is reported in Figure 4. It presents the typical features of an amorphous structure, with a broad peak centered around 2θ ~39°. This confirms the amorphous state of the investigated membrane, which, indeed, was produced by melt-spinning, in order to intentionally disrupt the crystalline order and render its structure amorphous (see Section 3).

**Figure 5:**
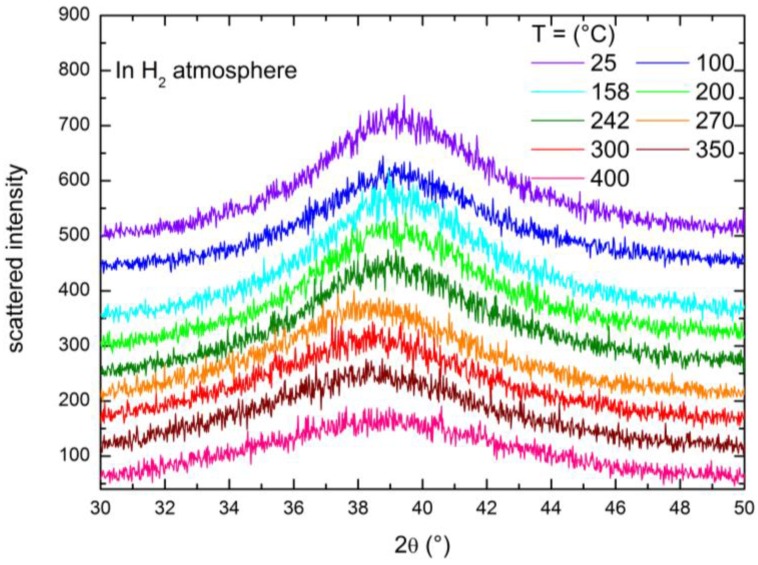
X-ray diffractograms measured on increasing temperature with the sample kept in a H_2_ atmosphere (p ~ 9 bar).

The temperature dependence of the structure of the Ni_32_Nb_28_Zr_30_Cu_10_ membrane was investigated by means of X-ray diffraction, heating the sample in a hydrogen (Figure 5) atmosphere between 25 and 400 °C in subsequent steps.

**Figure 6:**
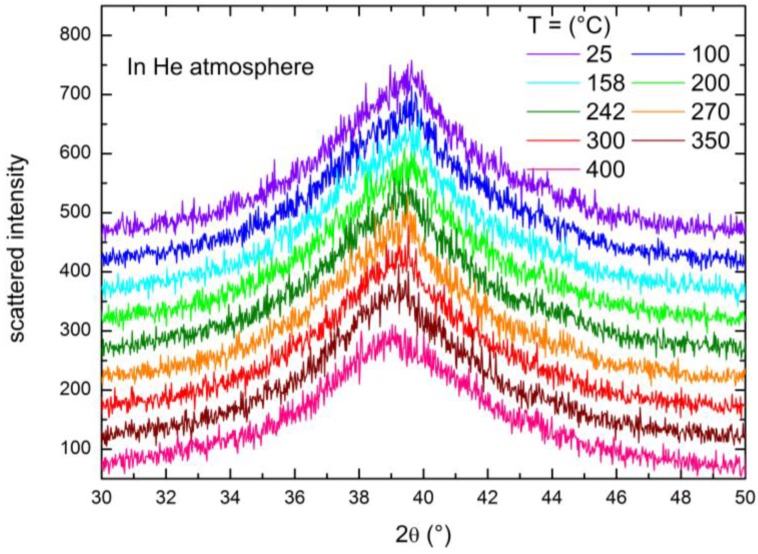
X-ray diffractograms measured with increasing temperature with the sample kept in a He atmosphere (p ~ 1 bar).

Heating the sample in H_2_ (Figure 5), the angular position of the peak remains unaltered between 25 and 242 °C, while abruptly, at 270 °C, the peak shifts to a lower angular position, which afterward stays fixed up to 400 °C. It is worth noting that the persistence of the single broad feature in the diffractogram even at high temperatures suggests that the amorphous nature of the membrane is retained up to the highest T investigated here (400 °C) and excludes a transition toward a crystalline state. For comparison, we conducted similar temperature-dependent XRD experiments on a Ni_32_Nb_28_Zr_30_Cu_10_ membrane under a helium atmosphere (p ~ 1 bar) and the results are displayed in Figure 6; in this case, no abrupt peak shift is detectable with the increasing temperature. This is a clear indication that the modifications occurring in the sample structure are mainly induced by the presence of hydrogen.

The angular position of the broad HTXRD peak is linked to the mean distance between atoms in the amorphous structure and the displacement towards lower 2θ values suggests an abrupt expansion of the structure at high temperatures.

To obtain a more quantitative picture, it must be noticed that the peak position at the highest intensity represents the main average interatomic distance, in either clusters or atoms in the Ni-rich amorphous matrix.

**Figure 7:**
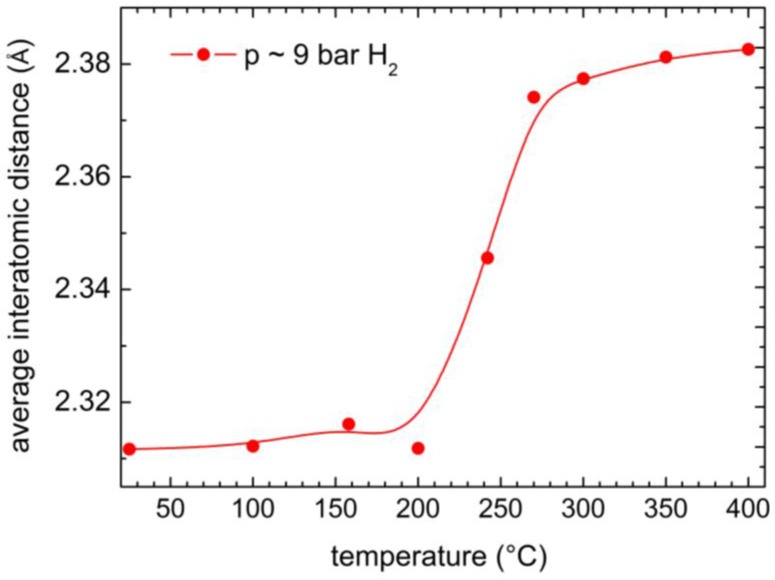
Average interatomic distances at the maxima of the X-ray diffraction peak (Figure 5) taken at 9 bar H_2_ pressure.

The 2θ values and the average interatomic distances obtained at the highest intensity are reported in Table 2, for both the X-ray diffraction patterns measured under H_2_ or He. In Figure 7, the average interatomic distances measured under hydrogen are plotted as a function of temperature: a significant expansion of the structure is observed between 200 and 300 °C, in agreement with the temperature (242 °C), where a change in the hydrogen absorption properties is detected by the previously reported pressure-composition isotherms. On the contrary, when heating in He, a normal thermal expansion, without abrupt changes, is detected (see Table 2).

From the present measurements, it is not possible to ascertain whether the entire icosahedra composing the amorphous structure or only some bonds between certain atoms expand in correspondence with the abrupt thermal expansion, but it is plausible that it may be due to an amorphous-to-amorphous phase transition. This transition would be induced by hydrogen, as it is not observed in the sample heated in He. An expansion of the Zr-Zr bond length induced by hydrogenation was already reported at room temperature [18], but in the present case, we are able to show that an abrupt and unexpected change occurs as the membrane is heated at high temperature under H_2_ atmosphere. Moreover, in the present work, we show that the two phases, at low and at high temperature, present different hydrogen sorption properties, with the lower temperature phase displaying a much higher value for the hydrogenation enthalpy.

## 3. Materials and Methods

Ni (purity 99.99% purity), Nb (99.8%), Zr (99.98%) and Cu (99.999%) were purchased from MilliporeSigma (Billerica, MA, United States). An alloy button of Ni_32_Nb_28_Zr_30_Cu_10_ was prepared by arc melting these metals in a purified argon atmosphere at AMES Laboratory Iowa, Ames, IA, USA. A melt spinning apparatus was used to fabricate the amorphous membranes at the CSIRO laboratory, Brisbane, Australia [16,19]. Typically the amorphous ribbons were ~30–70 µm thick and 30 mm wide [16,19].

The X-ray diffraction patterns were obtained using a PANalytical X’Pert pro θ-θ diffractometer (Almelo, The Netherlands). The ribbons were not coated with Pd. This instrument was equipped with a heating stage with Anton Paar XRK 900 (Graz, Austria). Hydrogen gas was introduced into the heating chamber via a home-made volumetric apparatus. The sample was placed inside the chamber and first evacuated using the turbo pump by Pfeiffer Vacuum model number TSH071E (Asslar, Germany).

Simultaneous TGA-DTA measurements were conducted by means of a Setaram Sensys Evolution 1200 TGA system (Caluire, France) [35] under a high purity argon flux (60 mL/min) at ambient pressure. For each experiment, a sample mass of ~10 mg was used. Different temperature rates, between 10 and 30 °C/min, were used for each sample in order to calculate the activation energy of the crystallization process.

The hydrogen absorption curves were recorded by the home-made Sieverts apparatus at Sapienza University of Rome described in Reference [23,36]. The experiments were performed on specimens with a mass of ~300 mg.

## 4. Conclusions

The hydrogen adsorption properties and the amorphous structure of a Ni_32_Nb_28_Zr_30_Cu_10_ membrane are studied by means of pressure-composition isotherms and XRD measurements, respectively. In the temperature range between 242 and 300 °C, an abrupt decrease of hydrogen absorption is observed; correspondingly, the hydrogen concentration measured at 7 bar is reduced from ~0.6 H/M at 242 °C to ~0.45 H/M at 300 °C. The X-ray diffractograms confirm that the membrane remains amorphous also at the highest investigated temperature (400 °C), and clearly indicate an abrupt increase of the mean interatomic distance from ~2.31 Å at 200 °C to ~2.38 Å occurs. This abrupt expansion is evidenced only when the samples are heated in a hydrogen atmosphere, while a much lower and non-abrupt thermal expansion is observed when samples are heated in an inert atmosphere. Here, for the first time, an abrupt change of the physical properties of amorphous membranes when heated in hydrogen at high temperatures is reported. Incidentally, these findings suggest that we should avoid extrapolations from properties measured close to room temperature.

## Figures and Tables

**Table 1:** The hydrogenation enthalpy, ΔH_hyd_,calculated for various hydrogen concentrations, H/M, values in the low (T ≤ 242 °C) and high temperature (T ≥ 300 °C) region.

ΔH_hyd_ (kJ/mol)	H/M = 0.30	H/M = 0.32	H/M = 0.34	H/M = 0.36	H/M = 0.38	H/M = 0.40
low T region	36 ± 1	31 ± 1	31 ± 1	29 ± 1	25 ± 1	21 ± 1
high T region	23 ± 1	21 ± 1	20 ± 1	18 ± 1	17 ± 1	15 ± 1

**Table 2:** The 2θ value and interatomic distances at various temperatures, measured under either H_2_ or He atmosphere.

T (°C)	Under Hydrogen (~9 bar)		Under Helium (~1 bar)	
	2θ	Interatomic Distance (Å)	2θ	Interatomic Distance (Å)
25	38.927	2.3117	39.287	2.2914
100	38.93	2.3122	39.235	2.2943
158	38.852	2.3161	39.221	2.2951
200	38.926	2.3118	39.223	2.295
242	38.343	2.3456	39.183	2.2973
270	37.866	2.3741	39.152	2.299
300	37.811	2.3774	39.127	2.3004
350	37.748	2.3812	39.059	2.3042
400	37.726	2.3826	39.207	2.2959

## References

[1] Ockwig N.W., Nenoff T.M. (2007). Membranes for Hydrogen Separation. Chem. Rev..

[2] Lu G.Q., Diniz da Costa J.C., Duke M., Giessler S., Socolow R., Williams R.H., Kreutz T. (2007). Inorganic membranes for hydrogen production and purification: A critical review and perspective. J. Colloid Interface Sci..

[3] Paglieri S., Way J.D. (2002). Innovations in palladium membrane research. Sep. Purif. Methods.

[4] Adhikari S., Fernando S. (2006). Hydrogen Membrane Separation Techniques. Ind. Eng. Chem. Res..

[5] Tosti S., Bettinali L., Violante V. (2000). Rolled thin Pd and Pd-Ag membranes for hydrogen separation and production. Int. J. Hydrog. Energy.

[6] Kanezashi M., Sano M., Yoshioka T., Tsuru T. (2010). Extremely thin Pd-silica mixed-matrix membranes with nano-dispersion for improved hydrogen permeability. Chem. Commun..

[7] Budd P.M., McKeown N.B. (2010). Highly permeable polymers for gas separation membranes. Polym. Chem..

[8] Yampolskii Y. (2012). Polymer Gas Separation Membranes. Macromolecules.

[9] Sanders D.F., Smith Z.P., Guo R., Robeson L.M., McGrath J.E., Paul D.R., Freeman B.D. (2013). Energy-efficient polymer gas separation membranes for a sustainable future: A review. Polymer.

[10] Razali M., Kim J.F., Attfield M., Budd P.M., Drioli E., Lee Y.M., Szekely G. (2015). Susteinable wastewater treatment and recycling in membrane manufacturing. Green Chem..

[11] Steward S.A. (1983). Review of Hydrogen Isotope Permeability through Metals.

[12] Sarker S., Chandra D., Hirscher M., Dolan M., Isheim D., Wermer J., Viano D., Baricco M., Udovic T.J., Grant D. (2016). Developments in the Ni-Nb-Zr Amorphous Alloy Membranes. Appl. Phys. A.

[13] Dolan M.D., Dave N.C., Ilyushechkin A.Y., Morpeth L.D., McLennan K.G. (2006). Composition and operation of hydrogen-selective amorphous alloy membrane. J. Membr. Sci..

[14] Hara S., Hatakeyma N., Itoh N., Kimura H.-M., Inoue A. (2003). Hydrogen permeation through amorphous Zr_36−*x*_Hf*_x_*Ni_64_ alloy membranes. J. Membr. Sci..

[15] Hara S., Sakaki K., Itoh N., Kimura H.-M., Asami K., Inoue A. (2000). An amorphous alloy membrane without noble metals for gaseous hydrogen separation. J. Membr. Sci..

[16] Hara S., Hatakeyma N., Itoh N., Kimura H.-M., Inoue A. (2002). Hydrogen permeation through palladium-coated amorphous Zr-M-Ni (M = Ti, Hf) alloy membranes. Desalination.

[17] Yamaura S.-I., Shimpo Y., Okouchi H., Nishida M., Kajita O., Kimura H., Inoue A. (2003). Hydrogen permeation characteristics of melt-spun Ni-Nb-Zr amorphous alloy membranes. Mater. Trans..

[18] Yamaura S.-I., Sakurai M., Hasegawa M., Wakoh K., Shimpo Y., Nishida M., Kimura H., Matsubara E., Inoue A. (2005). Hydrogen permeation and structural features of melt-spun Ni-Nb-Zr amorphous alloys. Acta Mater..

[19] Paglieri S.N., Pal N.K., Dolan M.D., Kim S.-M., Chien W.-M., Lamb J., Chandra D., Hubbard K.M., Moore D.P. (2011). Hydrogen permeability, thermal stability and hydrogen embrittlement of Ni-Nb-Zr and Ni-Nb-Ta-Zr amorphous alloy membranes. J. Membr. Sci..

[20] Kim S.-M., Chandra D., Pal N.K., Dolan M.D., Chien W.-M., Talekar A., Lamb J., Paglieri S.N., Flanagan T.B. (2012). Hydrogen permeability and crystallization kinetics in amorphous Ni-Nb-Zr alloys. Int. J. Hydrog. Energy.

[21] Adibhatla A., Dolan M.D., Chien W., Chandra D. (2014). Enhancing the catalytic activity of Ni-based amorphous alloy membrane surfaces. J. Membr. Sci..

[22] Kim S.-M., Chien W.-M., Chandra D., Pal N.K., Talekar A., Lamb J., Dolan M.D., Paglieri S.N., Flanagan T.B. (2012). Phase transformation and crystallization kinetics of melt-spun Ni_60_Nb_20_Zr_20_ amorphous alloy. J. Non-Cryst. Solids.

[23] Palumbo O., Brutti S., Trequattrini F., Sarker S., Dolan M., Chandra D., Paolone A. (2015). Temperature Dependence of the Elastic Modulus of (Ni_0.6_Nb_0.4_)_1−*x*_Zr*_x_* Membranes: Effects of Thermal Treatments and Hydrogenation. Energies.

[24] Fukubara M., Fujima N., Oji H., Inoue A., Emura A. (2010). Structures of the icosahedral clusters in Ni-Nb-Zr-H glassy alloys determined by first-principles molecular dynamics calculation and XAFS measurements. J. Alloys Compd..

[25] Fujima N., Hoshino T., Fukuhara M. (2013). Local structures and structural phase change in Ni-Zr-Nb glassy alloys composed of Ni_5_Zr_5_Nb_3_ icosahedral clusters. J. Appl. Phys..

[26] Jayalakshmi S., Fleury E. (2009). High temperature mechanical properties of rapidly quenched Zr_50_Ni_27_Nb_18_Co_5_ amorphous alloy. Met. Mater. Int..

[27] Jayalakshmi S., Park S.O., Kim K.B., Fleury E., Kim D.H. (2007). Studies on hydrogen embrittlement in Zr- and Ni-based amorphous alloys. Mater. Sci. Eng. A.

[28] Yamaura S.-I., Simpo Y., Okouchi H., Nishida M., Kajita O., Inoue A. (2004). The Effect of Additional Elements on Hydrogen permeation Properties of Melt-spun Ni-Nb-Zr amorphous alloys. Mater. Trans..

[29] Palumbo O., Trequattrini F., Pal N., Hulyalkar M., Sarker S., Chandra D., Flanagan T., Dolan M., Paolone A. (2017). Hydrogen absorption properties of amorphous (Ni_0.6_Nb_0.4−*y*_Ta_y_)_100−*x*_Zr*_x_* membranes. Prog. Nat. Sci..

[30] Jayalakshmi S., Choi Y.G., Kim Y.C., Kim Y.B., Fleury E. (2010). Hydrogenation properties of Ni-Nb-Zr-Ta amorphous ribbons. Intermetallics.

[31] Jayalakshmi S., Vasantha V.S., Fleury E., Gupta M. (2012). Characteristics of Ni–Nb-based metallic amorphous alloys for hydrogen-related energy applications. Appl. Energy.

[32] Conic D., Gradisek A., Radakovic J., Iordoc M., Mirkovic M., Cebela M., Batalovi K. (2015). Influence of Ta and Nb on the hydrogen absorption kinetics in Zr-based alloys. Int. J. Hydrogen Energy.

[33] Hao S., Sholl D.S. (2010). Comparison of first principles calculations and experiments for hydrogen permeation through amorphous ZrNi and ZrNiNb films. J. Membr. Sci..

[34] Karger B.L., Snyder L.R., Horvath C. (1973). An Introduction to Separation Science.

[35] Palumbo O., Paolone A., Rispoli P., Cantelli R., Autrey T. (2010). Decomposition of NH_3_BH_3_ at sub-ambient pressures: A combined thermogravimetry–differential thermal analysis–mass spectrometry study. J. Power Sources.

[36] Palumbo O., Trequattrini F., Vitucci F.M., Bianchin A., Paolone A. (2015). Study of the hydrogenation/dehydrogenation process in the Mg-Ni-C-Al system. J. Alloys Compd..

